# Clinical risk factors for age-related macular degeneration: a systematic review and meta-analysis

**DOI:** 10.1186/1471-2415-10-31

**Published:** 2010-12-13

**Authors:** Usha Chakravarthy, Tien Y Wong, Astrid Fletcher, Elisabeth Piault, Christopher Evans, Gergana Zlateva, Ronald Buggage, Andreas Pleil, Paul Mitchell

**Affiliations:** 1 Centre for Vision Science, Queen's University Belfast, Northern Ireland, UK; 2 Singapore Eye Research Institute, National University of Singapore, Singapore; 3 Centre for Eye Research Australia, University of Melbourne, Royal Victorian Eye and Ear Hospital, Melbourne, Australia; 4 Dept of Epidemiology & Population Health, London School of Hygiene & Tropical Medicine, London, UK; 5 Mapi Values, Boston, Massachusetts, USA; 6 Pfizer Inc, New York, New York, USA; 7 Novartis, East Hanover, New Jersey, USA; 8 Centre for Vision Research, Westmead Millennium Institute, University of Sydney, Sydney, Australia

## Abstract

**Background:**

Age-related macular degeneration (AMD) is the leading cause of blindness in Western countries. Numerous risk factors have been reported but the evidence and strength of association is variable. We aimed to identify those risk factors with strong levels of evidence which could be easily assessed by physicians or ophthalmologists to implement preventive interventions or address current behaviours.

**Methods:**

A systematic review identified 18 prospective and cross-sectional studies and 6 case control studies involving 113,780 persons with 17,236 cases of late AMD that included an estimate of the association between late AMD and at least one of 16 pre-selected risk factors. Fixed-effects meta-analyses were conducted for each factor to combine odds ratio (OR) and/or relative risk (RR) outcomes across studies by study design. Overall raw point estimates of each risk factor and associated 95% confidence intervals (CI) were calculated.

**Results:**

Increasing age, current cigarette smoking, previous cataract surgery, and a family history of AMD showed strong and consistent associations with late AMD. Risk factors with moderate and consistent associations were higher body mass index, history of cardiovascular disease, hypertension, and higher plasma fibrinogen. Risk factors with weaker and inconsistent associations were gender, ethnicity, diabetes, iris colour, history of cerebrovascular disease, and serum total and HDL cholesterol and triglyceride levels.

**Conclusions:**

Smoking, previous cataract surgery and a family history of AMD are consistent risk factors for AMD. Cardiovascular risk factors are also associated with AMD. Knowledge of these risk factors that may be easily assessed by physicians and general ophthalmologists may assist in identification and appropriate referral of persons at risk of AMD.

## Background

Age-related macular degeneration (AMD) is the leading cause of blindness among people aged 55 years and older in the U.S and other Western countries [1-3]. Late stage AMD includes two morphological sub-types: neovascular AMD and geographic atrophy [4,5]. Population studies indicate that neovascular AMD accounts for two thirds of late AMD cases, and 90% of blindness from AMD [6]. Left untreated, neovascular AMD results in severe visual impairment with an average loss of around 4 lines of visual acuity within 2 years of disease onset [7]. Patients with geographic atrophy also develop visual loss although this tends to be more gradual.

With the introduction of new and effective treatments for neovascular AMD, there is a strong rationale for early identification of persons at highest risk of progression to the late stages as timely treatment given at the onset of neovascular AMD will lead to better visual outcomes [8-11]. In this regard, a number of major risk factors for AMD have been identified, including genetic (e.g., complement factor H polymorphisms), demographic (e.g., ethnicity), nutritional (e.g., antioxidant vitamins, dietary fats or fish), lifestyle (e.g., smoking), medical (e.g., cardiovascular risk factors), environmental (e.g., sun exposure), and ocular factors [12-16]. However, the evidence and strength of association remain variable in the literature. Furthermore, a number of these risk factors (e.g., diet and genetic factors) are not easily measured in routine clinical practice [17-21]. While ocular clinical signs such as drusen and pigmentary irregularities are important markers for progression to late AMD [22], the skills required for an appropriate retinal evaluation to be performed followed by the interpretation of the severity of the signs to make a meaningful judgement of risk observed are limited to those with retinal specialist knowledge. The impending explosion in immunomodulatory pharmacotherapies which are in currently in early phase clinical trials constitute another important reason for non-specialist clinicians and general ophthalmologists to be able to refer persons at very high risk of development of late AMD. Therefore it was our view that more precise estimates of risk for factors that could be assessed through routine history taking would be of value for appropriate counselling and referral.

## Methods

### Selection of Risk factors

We performed a systematic review and meta-analysis of a selection of risk factors for late AMD (neovascular AMD and geographic atrophy). Initially, we scrutinized in detail the literature on late AMD to identify all possible risk factors. The initial search yielded 73 possible risk factors (Table 1). Following review by an expert panel (UC, AF, PM, TYW), 16 factors that were considered to be readily measured in nonspecialist settings were selected for the full systematic review. We did not address ocular or genetic risk factors as these require either specialist skills to conduct the retinal examination or access to laboratory resources and genetic expertise. We also excluded nutrition as an estimation of the nutrition status through dietary questionnaires is also a specialised field [23].

**Table 1:** Potential 73 Risk Factors for Late Age-related macular degeneration, Identified in the Initial Review

• **Ocular factors (n = 15)**
• Nuclear opacity
• Cortical opacity
• Pterygium
• Lens opacity
• Horizontal cup-to-disc ratio
• Fellow eye
• Iris color*^†^
• Eye disease*
• Cataract/Cataract surgery*^†^
• Arcus cornea
• Arterioar-to-venular ratio
• Frekling
• Spherical equivalents
• Eye glasses for distance vision
• *Family history of AMD*^†^
• **Cardiovascular factors (n = 7)**
• Atherosclerosis
• History of CVD and cerebrovascular disease*^†^
• *Serum Total Cholesterol level*^*† *^*and Serum HDL Cholesterol level*^*†*^
• Hypertension*^†^
• Plasma antioxidants
• **Other medical conditions or marker (n = 8)**
• Biochemical variables* including serum albumin, *C-Reactive Protein*,*^† ^*plasma fibrinogen*,^† ^and *serum triglyceride*^†^)
• Bone mineral density
• Diabetes*^†^
• Arthritis
• Menopause
• **Diet (n = 11)**
• Dietary intake
• Fat*
• Animal fat*
• Vegetable fat*
• Linoleic acid
• ∞-3 fatty acids EPA/DHA
• Antioxidants*
• Saturated fat
• Monounsaturated fat
• Polyunsaturated fat
• Trans-unsaturated fat
• **Medications (n = 9)**
• Birth control use
• Diuretics use
• Antacid use
• Antihypertensive medication use
• Anti-inflammatory drug use
• Hydrochlorothiazide use
• Hormone replacement therapy
• Hormones (women)
• Hypnotics/sedative
• **Life style (n = 3)**
• Smoking*^†^
• Alcohol consumption
• Physical activity level
• **Light and other exposures (n = 3)**
• Place of birth
• Solar radiation/outdoor exposure*
• Chemical exposure
• **Genetics (n = 9)**
• fibulin 5
• CST3
• CX3CR
• TLR4
• VEGF
• LRP6
• MMP9
• HLA family of genes
• CFH
• **Demographics (n = 8)**
• Gender*^†^
• Age*^†^
• Race/ethnicity*^†^
• Education*
• Weight/Body mass index*^†^
• Waist circumference
• Height
• Marital status

### Data Sources

Searches were conducted in Medline and Cochrane databases using these terms: macular degeneration OR age-related macular degeneration OR age-related maculopathy AND gender OR age OR race OR ethnicity OR iris colour OR diabetes OR cardiovascular OR cerebrovascular OR hypertension OR smoking OR cataract surgery OR family history OR body mass index OR cholesterol OR fibrinogen OR C-reactive protein OR triglyceride.

### Studies and Participants

Prospective cohort, case-control, or cross-sectional studies, were included if they incorporated an estimate of association [odds ratio (OR) or relative risk (RR)] between late AMD and at least one of the 16 risk factors. Non-English language articles were not included because, after a preliminary assessment, we did not identify any that fitted our inclusion criteria. Abstracts and unpublished studies were also not included.

For articles that passed initial screen, additional criteria were applied for inclusion in the meta-analysis: 1) estimates for association with neovascular AMD and geographic atrophy were excluded when concomitantly presented as late AMD to avoid double counting; 2) for three epidemiological studies that examined the risk of late AMD (i.e., Beaver Dam [24-26], Blue Mountains [24-26], and Rotterdam [25,26]) both the original estimates and pooled estimates of the three studies exist. Therefore, to avoid double counting of these studies, only the pooled estimate was retained. We did not include studies that reported only the unadjusted results (i.e., crude ORs). All studies included adjusted for age; when studies reported more than one adjusted estimate, the multivariate adjusted estimate was selected (i.e., if a study reported either age adjusted or age, gender and smoking adjusted results, the latter was chosen).

### Outcome Measures

The primary outcome measure was late AMD, or each type of late AMD if the overall results for late AMD were not presented.

### Study Selection

Searches were completed by 28 February 2007 and yielded 295 references. The abstracts of these articles were independently reviewed by two researchers (LP and CE) and relevant data (e.g., study design, risk factor, type of AMD) was recorded on a study-specific abstraction form. Full-text articles were then obtained based on the initial screening of abstracts and the data extraction form was completed. Any disagreements between the two reviewers in the abstract review or following selection of articles for full text review were resolved by discussion. The bibliographies of the full text articles that were reviewed were searched for relevant references.

**Figure 1:**
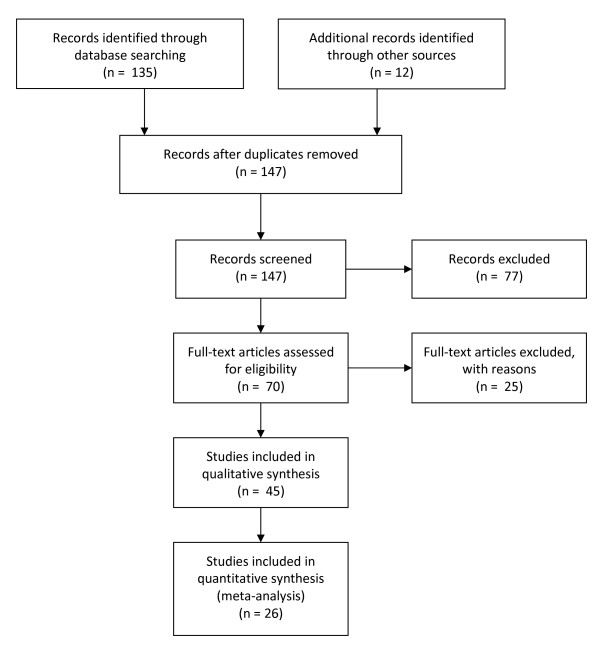
**Study selection process**.

In all, 128 unduplicated references were initially identified as being of potential relevance (the search was repeated in November 2008 and seven additional articles published in 2008 were included [27-32]), with 12 additional articles retrieved from the reference lists. From the total of 147 articles, 70 fulfilled all eligibility criteria and were selected for meta-analysis. One study was excluded since the outcome was determined by clinical criteria rather than retinal photography. In addition, 24 articles were excluded since authors reported progression for early AMD as well as late AMD. In the final pool, 45 articles were retained for the meta-analyses including articles with results from 18 prospective studies [24,26,33-49], and cross-sectional studies [12,25,28,30,31,50-63], and 8 case control studies (Figure 1) [50,64-70].

### Data Synthesis

We used Comprehensive Meta-Analysis software version 2 for all meta-analyses. An overall meta-analysis was performed for each risk factor if there were two or more risk estimates irrespective of study design. In addition, since the study design could influence the risk estimate, meta-analyses were performed for each major type of study within each risk factor. We conducted fixed-effects and random-effects meta-analyses to combine these outcomes across included studies by study design, estimating overall raw point estimates of each risk factor and their associated 95% confidence intervals (CI). The selection of fixed-effects vs. random-effects models was based on the reported I-squared value and associated p-value. If the p-value was < 0.10 then random-effects was chosen, otherwise fixed-effects was selected, except when there was clear heterogeneity based on individual study results and in these instances the more conservative random-effects was selected. Forest plots were used to graphically present the significant findings. Funnel plots were produced to explore the potential for publication bias.

## Results

In total, data from 18 prospective, cross-sectional studies and 8 case control studies [12,24-26,28,30,31,33-47,49-54,57-73] were included in the final analysis, contributing a sample of some 94,058 patients (ranging from 261 to 22,071) including 3,178 (ranging from 8 to 776) late AMD cases. Table 2 summarizes the characteristics of the 18 prospective and cross-sectional studies from which estimates were included in the meta-analysis. Funnel plots were reviewed and no evidence of publication bias was observed.

**Table 2:** Summary of characteristics of the 18 prospective and cross sectional studies

STUDY NAME	LOCATION	DATE OF STUDY	POPULATION	NUMBER OF LATE AMD SUBJECTS	AGE RANGE	% MALE	METHOD OF FUNDUS CAPTURE	METHOD OF CLASSIFICATION
AREDS [64]	USA	1992-1998	4,519	776	60-80	NA	Retinal photo	WARMGS

Baltimore Eye Study*[91]	USA	1985-1988	4,396	48	40+	NA	Retinal photo	International AMD

Barbados Eye Study*[42]	Barbados	1988-1992	2,374	12	40-84	43	Retinal photo	Described in paper

Beaver Dam Eye Study[24-26,39,47,57,92]	USA	1988-1990	4,926	72	43-86	44	Retinal photo	WARMGS

Blue Mountains Eye Study*[24-26,35,43,46]	Australia	1992-1994	3,654	65	49+	43	Retinal photo	WARMGS

Cardiovascular Health Study[40]	USA	1989-1990	5,201	35	65+	60	Retinal photo	WARMGS

Copenhagen City Study*[33,49]	Denmark	1986-1988, 2000-2002	946	112	60-80	49	Retinal photo	Described in paper

European Study of Eye Disease (EUREYE)[12]	Europe	2000-2003	4,752	158	Aged 65+	45	Retinal photo	International AMD

Funagata Study[30]	Japan	2000-2002	3,676	8	35+	51	Retinal photo	WARMGS

Los Angeles Latino Eye Study (LALES) *[53,54,61]	USA	1997-2002	5,875	25	40+	42	Retinal photo	WARMGS

Multi-ethnic Study of Atherosclerosis (MESA)[41,59]	USA	2002-2004	6,176	27	45-85	48	Retinal photo	WARMGS

NHANES III*[58]	USA	1998-1994	8,270	54	40+	47	Retinal photo	WARMGS

PHS[34,44]	USA	1982-1989	22,071	64	40+	100	Retinal photo	WARMGS

Progression of Age-related Macular Degeneration Study (PARMDS)[45]	USA	1989-1998	261	NA	60+	NA	Retinal photo	Modification of the Age-Related Eye Disease Study Grading system

Pathologie Occulaires Liees a l'age (POLA) *[51,52]	France	2002, 2006	2,183	41	60+	43	Retinal photo	Fundus photo

Proyecto VER*[93]	USA	1997-1999	2,780	15	50+	39	Retinal photo	WARMGS

Rotterdam Study*[25,26,36,62,94]	Netherlands	1990-1993	6,251	104	55+	40	Retinal photo	WARMGS

Singapore Malay Eye Study (SiMES)[28,31]	Singapore	1992	3,280	23	40-80	52	Retinal photo	WARMGS

WARMGS: Wisconsin Age-Related Maculopathy Grading System; * Studies included in the meta-analyses

* From article referenced

Note: Case control studies were not included in this table

The findings for each risk factor except age are summarized in Table 3 and discussed separately in the following sections.

**Table 3:** Summary Results from the Meta-analysis

RISK FACTOR	PROSPECTIVE		CROSS-SECTIONAL		CASE CONTROL	
	**n**	**Overall estimate**	**n**	**Overall estimate**	**n**	**Overall estimate**

Gender (female)	2	1.01 (0.89 - 1.16)	2	1.06 (0.78 - 1.44)	2	1.00 (0.83 - 1.21)

Race (white vs. other)	3	0.91 (0.49 - 1.69)	3	1.09 (0.09 - 13.56)*	1	4.2 (2.23 - 8.00)

Family History*	0		1	3.95 (1.35 - 11.54)	2	6.18 (0.98 - 38.90)*

Cataract surgery*	4	3.05 (2.05 - 4.55)	2	1.59 (1.08 - 2.34)	3	1.54 (1.24 - 1.91)

Iris colour*	5	0.98 (0.72 - 1.32)	4	0.88 (0.65 - 1.17)	2	0.60 (0.12 - 2.98)*

Body Mass Index*	9	1.28 (0.98 - 1.67)*	10	1.21 (0.97 - 1.53)	2	1.52 (1.15 - 2.00)

Smoking*	5	1.86 (1.27 - 2.73)	7	3.58 (2.68 - 4.79)	6	1.78 (1.52 - 2.09)

Hypertension*	4	1.02 (0.77 - 1.35)	6	1.15 (0.88 - 1.51)	3	1.48 (1.22 - 1.78)

Diabetes*	3	1.66 (1.05 - 2.63)	3	1.09 (0.61 - 1.92)	1	0.55 (0.06 - 4.87)

Cardiovascular Disease*	7	1.22 (0.92 - 1.63)	9	1.12 (0.86 - 1.47)	6	2.20 (1.49 - 3.26)*

Cerebrovascular Diseases	2	1.54 (0.82 - 2.90)	5	1.10 (0.69 - 1.75)	0	

Serum Cholesterol (Total)	4	0.99 (0.95 - 1.03)	5	0.94 (0.84 - 1.04)	1	4.66 (1.35 - 16.11)

Serum Cholesterol (HDL)	3	1.00 (0.97 - 1.02)	5	1.06 (0.80 - 1.39)	1	3.35 (0.92 - 12.23)

Serum Triglycerides	2	1.00 (0.77 - 1.30)	3	1.08 (0.89 - 1.30)	1	0.90 (0.25 - 3.24)

Plasma Fibrinogen*	1	1.03 (0.81 - 1.32)	3	1.45 (1.22 - 1.73)	0	

n = number of estimates entered in the models; *Figure is displayed

* Estimates from random effects models. All other estimates are fixed effects.

### Age

**Figure 2:**
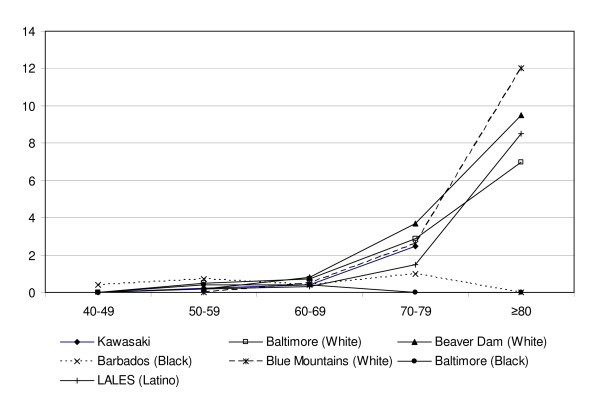
**Prevalence of late AMD by age (adapted from Varma et al**[61]**and Kawasaki et al**[19]).

All studies found a strong association with increasing age (Table 4 and Figure 2) [31,74].

**Table 4:** Prevalence of late AMD by age (Adapted from Varma et al68 and Kawasaki et al29)

Age	Los Angeles	Baltimore	Blue Mountains	Beaver Dam	Baltimore	Barbados	SiMES
Group	Latino	White	White	White	Black	Black	Asian
(yrs)	[% (CI)]	[% (CI)]	[% (CI)]	[% (CI)]	[% (CI)]	[% (CI)]	[%]
40 - 49	0.0	0.0			0.0	0.4 (0.0 - 0.8)	0
50 - 59	0.2 (0.0 - 0.4)	0.5 (0.0 - 1.1)	0.0	0.2 (0.0 - 0.4)	0.4 (0.0 - 0.8)	0.7 (0.2 - 1.2)	0.21
60 - 69	0.3 (0.0 - 0.6)	0.7 (0.1 - 1.3)	0.5 (0.1 - 0.8)	0.8 (0.3 - 1.3)	0.4 (0.0 - 1.0)	0.4 (0.0 - 0.8)	0.39
70 - 79	1.5 (0.5 - 2.5)	2.9 (1.5 - 4.4)	2.6 (1.6 - 3.6)	3.7 (2.5 - 4.9)	0.0	1.0 (0.0 - 2.0)	2.49
≥ 80	8.5 (3.5 - 13.5)	7.0 (2.0 - 12.0)	12.0 (8.7 - 15.4)	9.5 (6.2 - 12.8)	0.0	0.0	

### Gender

**Figure 3:**
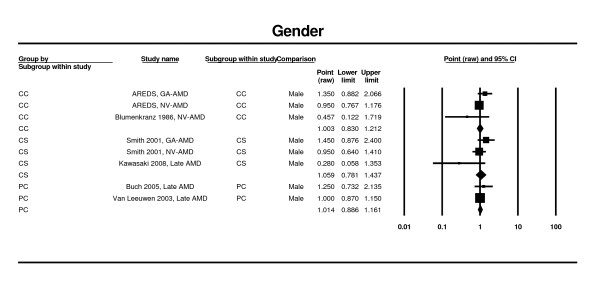
**Pooled odds ratio for late AMD by gender (female vs. male)**.

Two estimates from two case control studies [64,65], two estimates from cross-sectional studies [25,30], and two prospective studies [27,75] contributed to this meta-analysis. Findings from this analysis suggest that there is no significant association between female gender and late AMD. In the case control studies, the overall OR for female gender was 1.00 (95% CI 0.83 - 1.21), in the cross-sectional studies, it was 1.06 (95% CI 0.78 - 1.44) (Figure 3). In the prospective studies, it was 1.01 (95% CI 0.89 - 1.16). Since the authors had not provided the confidence intervals, one study could not be included in the model [49].

### Race/ethnicity

**Figure 4:**
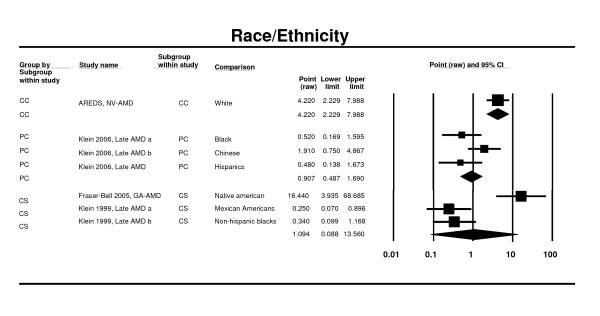
**Pooled odds ratio for late AMD and by race/ethnicity (whites vs. other races/ethnicities)**.

One case control study (AREDS) [64], two cross sectional studies (NHANES III and LALES) [53,58] and two prospective studies (MESA, CHS) [41,76] contributed to this analysis. All studies were based in the US population. In the MESA Study [41], there were no significant differences between whites (European origin), Asians (i.e. predominantly Chinese ancestry), Blacks (African Americans) or Hispanics in late AMD prevalence. In the NHANES III [58], there were no significant differences between Non-Hispanic White, Non-Hispanic Black (OR 0.34, 95%CI 0.10 - 1.18) and Mexican Americans (OR 0.25, 95% CI 0.07 - 0.90). In the LALES [53], individuals of Native American ancestry were nearly 15 times more likely to have geographic atrophy (95% CI 1.8 - 12.6) than Latinos. The OR was 0.91 (0.49 - 1.69) in prospective cohort studies and 1.09 (0.09 - 13.56) in cross-sectional studies for whites versus other races/ethnicities (Figure 4). An additional study, the Baltimore Eye Study [77], was identified; however, the use of differing nomenclatures in the Baltimore Eye Study to identify racial types (e.g., African American, Chinese, Mexican American) prevented its inclusion in the meta-analysis.

### Family history

**Figure 5:**
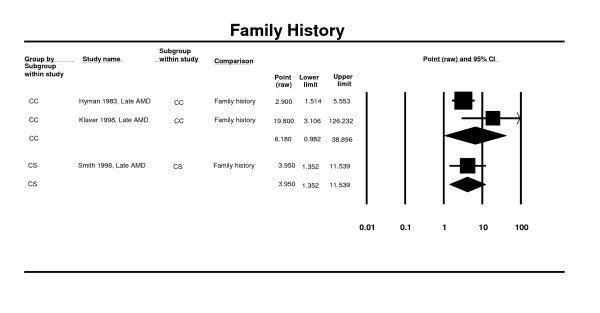
**Pooled odds ratio for late AMD by family history (presence or absence)**.

Two case control studies were included in the meta-analysis [68,69]. Findings from the meta-analysis show an insignificant association between family history and late AMD (OR 6.18; 95% CI 1.94 - 6.61). This was supported by findings from a cross-sectional study (OR 3.95. 95% CI 1.35 - 11.54) (Figure 5) [60].

### Cataract surgery

**Figure 6:**
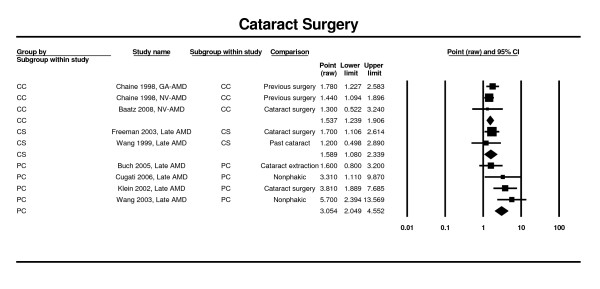
**Pooled odds ratio for late AMD by history of previous cataract surgery**.

Estimates from three prospective cohort studies (i.e., Copenhagen, Blue Mountains, and Beaver Dam) [24,27,35,38] at five-years and ten-years, and from five cross-sectional studies (Salisbury Eye Evaluation, Proyecto VER, Baltimore Eye Survey, and Blue Mountains) [63,78], were analyzed. Only two case control studies [50,66] were found. Analysis of the prospective cohort studies showed that previous cataract surgery is a strong risk factor for neovascular AMD (RR 3.05; CI 2.05 - 4.55). This finding is supported by the results of the meta-analysis of the cross-sectional studies (OR = 1.59; CI 1.08 - 2.34) (Figure 6).

### Smoking

**Figure 7:**
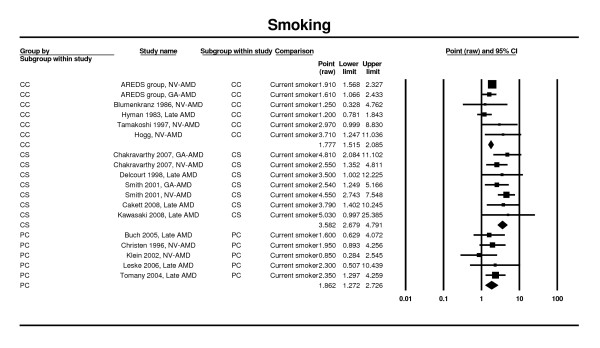
**Pooled odds ratio for late AMD by smoking status (current vs. never)**.

Estimates were reported from six prospective cohort studies [26,33,34,42,79], five case control studies [64,65,67,68,70] and five cross-sectional studies [12,25,28,30,51] contributed to the meta-analysis. Significant increases in AMD risk were seen in all the meta-analyses for current versus never smokers. The OR for case control studies was 1.78 (95% CI 1.52 - 2.09), and that from cross-sectional studies was 3.58 (95% CI 2.68 - 4.79). The RR obtained through analysis of prospective cohort studies was 1.86 (95% CI 1.27 - 2.73) (Figure 7).

### Iris colour

**Figure 8:**
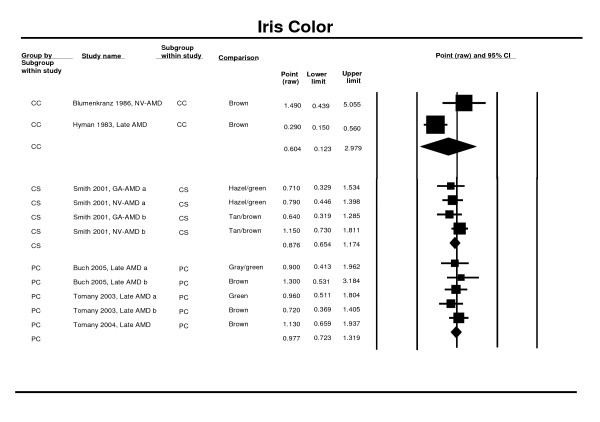
**Pooled odds ratio for late AMD by iris color (brown vs. blue eyes)**.

Data from three prospective cohort studies [26,27,47], from two control studies [65,68] and a pooled estimate from a cross sectional study [25] were used in the meta-analysis. Two studies were not used because the authors had not provided the confidence interval of the estimate [37,64], or because the data had already been considered within a pooled analysis [58]. The meta-analysis of the prospective cohort and cross-sectional studies suggests that darker iris pigmentation (brown vs. blue eyes) is protective, but the overall results were not significant (OR 0.88; 95% CI 0.65 - 1.17 cross-sectional studies and RR 0.98; 95% CI: 0.72 - 1.32 for prospective studies). The case control results support this finding (0.60; 95% CI 0.12 - 2.98) (Figure 8).

### Body Mass Index (BMI)

**Figure 9:**
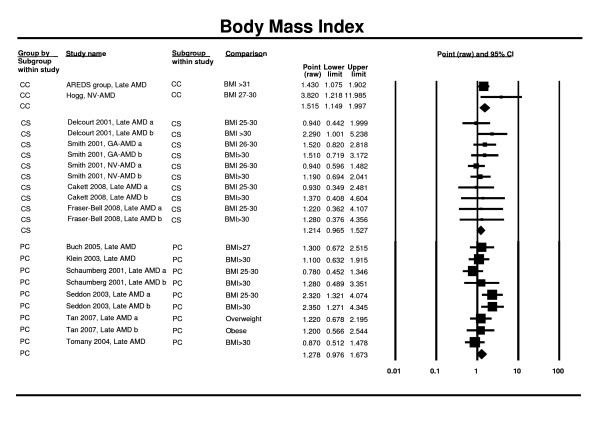
**Pooled odds ratio for late AMD by body mass index (obese vs. non-obese)**.

Seven prospective cohort studies (Copenhagen, CHS, PHS, PARMDS, Beaver Dam, Rotterdam, Blue Mountains) [26,33,44-46,80] and six cross-sectional studies (POLA, Beaver Dam, Rotterdam, Blue Mountains, LALES, SiMES) [25,28,52,54] contributed to the meta-analysis. Analysis of the prospective studies showed an adverse effect of being overweight/obese and the risk of late AMD (RR 1.28; 95% CI 0.98 - 1.67). Cross-sectional study findings were in the same direction but did not reach statistical significance (OR 1.21; 95% CI 0.97 - 1.53). However, the two case control studies [64,67] did achieve statistical significance (OR 1.52; 95% 1.15 - 2.00) (Figure 9).

### Hypertension

**Figure 10:**
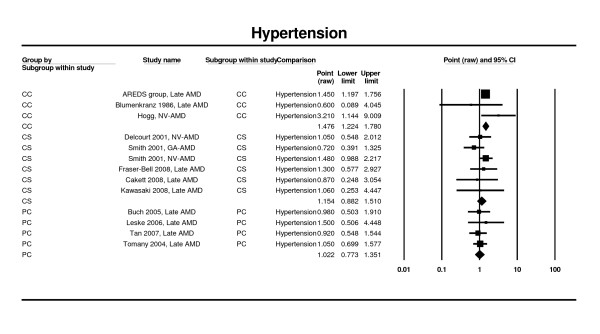
**Pooled odds ratio for late AMD by hypertension (presence or absence)**.

Estimates from five prospective cohort studies [26,27,42,46], three cases control studies [64,65,67], and from seven cross-sectional studies [25,28,30,52,54] contributed to this analysis. None of the analyses showed statistically significant associations (prospective cohort RR 1.02; 95% CI 0.77 - 1.35; cross-sectional studies OR 1.15; 95% CI: 0.88 - 1.51). The three case control studies did identify a significant association between presence of hypertension and late AMD (OR 1.48; 95% CI 1.22 - 1.78) (Figure 10).

### Diabetes

**Figure 11:**
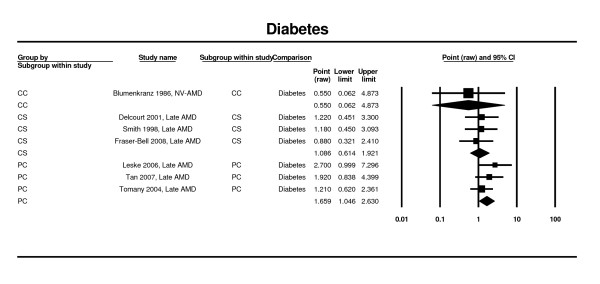
**Pooled odds ratio for late AMD by diabetes (presence or absence)**.

Estimates from four prospective cohort studies [24,42,46] and two cross-sectional estimates [52,54,60] contributed to the meta-analysis. Based on the data from the prospective cohort studies, the presence of diabetes was associated with an increased risk of late AMD (RR 1.66; 95% CI 1.05 - 2.63). In the cross-sectional estimates that used data from two studies, associations were nonsignificant (OR 1.09; 95% CI 0.61 - 1.92). Only one case control study [65] showed nonsignificant association with diabetes (OR 0.55; 95% CI 0.06 - 4.87) (Figure 11).

### History of Cardiovascular Disease

**Figure 12:**
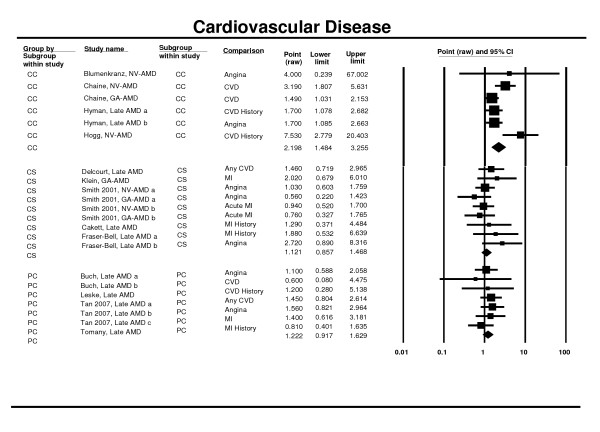
**Pooled odds ratio for late AMD by cardiovascular disease (presence or absence)**.

Five prospective cohort studies (including pooled estimates) [26,33,42,46] four case control studies [65-68] and seven cross-sectional studies [28,52,54,58] (including pooled estimates [25]) contributed to the meta-analysis of history of cardiovascular disease (self or medical report) and late AMD. As previously mentioned, some estimates [49,81] were not retained in the analysis. No significant association was found in the prospective cohort and the cross-sectional studies (RR 1.22, 95% CI 0.92 - 1.63 and OR 1.12; 95% CI 0.86 - 1.47). A significant association was observed in the case control studies, with around double the odds of late AMD in individuals with cardiovascular disease (OR 2.20; 95% CI 1.48 - 3.26) (Figure 12).

### History of Cerebrovascular Diseases

**Figure 13:**
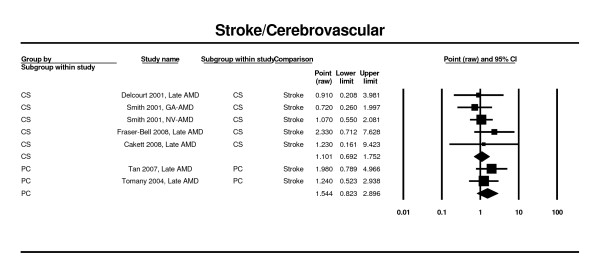
**Pooled odds ratio for late AMD by cerebrovascular disease (presence or absence)**.

Three prospective cohort studies [26,46] and six cross-sectional studies [25,28,52,54] were included in the meta-analysis of history of cerebrovascular diseases and late AMD (Figure 13). No significant associations were found (RR 1.54; 95% CI 0.82 - 2.90 and OR 1.10, 95%CI 0.69 - 1.75 for the prospective and the cross-sectional studies, respectively).

### Biological markers

Five prospective cohort studies [26,33,40,46] (including one pooled estimate), and six cross-sectional studies [28,30,52] (including one pooled estimate [25]), one case control [67] of serum total cholesterol and of serum high-density lipoprotein cholesterol contributed to the meta-analysis (data not shown). Three studies [49,57,82] were not included in the meta-analysis. The pooled odds ratios and relative risks were non-significant for both serum cholesterol measures.

Neither the two prospective cohort studies [27,46] (RR 1.0; 95% CI 0.77 - 1.30) nor the cross-sectional studies[28,52,60] (OR 1.08; 95% CI 0.89 - 1.30), found an association between serum triglycerides and late AMD (data not shown).

**Figure 14:**
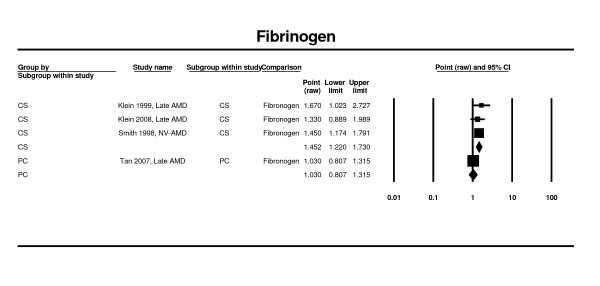
**Pooled odds ratio for late AMD by plasma fibrinogen**.

A significant increase in risk of late AMD with increased plasma fibrinogen was observed in two cross-sectional studies [58-60], (OR 1.45; 95% CI 1.22 - 1.73). There was only one estimate from a prospective study [46] and it did not support this finding (OR 1.03; 95% CI 0.81 - 1.32) of significance in the cross-sectional studies (Figure 14).

None of the studies which measured C-reactive protein [36] were eligible for inclusion in our meta-analysis.

## Discussion

Identifying patients at high risk of late AMD, particularly neovascular AMD, is important from both public health and clinical perspectives as this would facilitate detection of disease before the onset of irreversible visual loss enabling earlier intervention. Of the 16 risk factors identified in our systematic review and meta-analysis, age, current smoking, cataract surgery, and potentially family history were strongly and consistently associated with late AMD. All of these are easily assessed through discussions with patients and do not entail a lengthy medical history taking or laboratory evaluations. Other significant factors with a lower strength of association (risk estimates generally 1.5 or less) were BMI, hypertension, a history of cardiovascular disease and plasma fibrinogen. All of these factors are associated with cardiovascular disease and are also likely to be measured and monitored in the primary care setting.

Our meta-analysis confirmed the increased risks of late AMD associated with advancing age (especially for the oldest age groups of 80 years and over), current cigarette smoking, and previous cataract surgery. The relationship of cataract surgery and late AMD was previously reported in a pooled analysis of 3 studies in different continents [26]. Although this association could reflect shared risk factors and the fact that both are diseases that affect the ageing eye, there is concern that surgery may predispose the operated eye to the development of neovascular AMD. We further confirmed the association with family history of AMD, consistent with the growing recognition of major genes involved in AMD (e.g., complement factor H and C3, LOCS 3877, HTRA 1) [21,83-86].

Vascular diseases, including myocardial infarction, stroke, angina, and hypertension are thought to be pathogenic factors for the development of late AMD. Our meta-analysis showed that while the magnitude of the ORs were inconsistent across studies, the pooled estimates for case-control studies were statistically significant for both cardiovascular disease (OR 2.20; 95% CI 1.49 - 3.26) and hypertension (OR 1.48, 95% CI 1.22 - 1.78). The association of diabetes and late AMD is also less consistent, and while prospective studies showed a significant association, this was not significant in the cross-sectional or case-control studies. The relationship between higher BMI and late AMD could be explained by shared risk factors (e.g., hypertension), or potential unmeasured confounders (e.g., nutritional factors). Our analysis also indicated that, based only on US data, people of European origin were at increased risk compared to other ethnic groups but at present the evidence for risks associated with other specific ethnic groups is inadequate. These are areas of future research.

Our study has several limitations. Although we selected only studies that reported some adjustment for confounding factors, we could not ascertain the appropriateness or completeness of adjustment in the studies. Second, the data included in some studies may have been too crude and also subject to measurement error. For example, a 'history of cardiovascular disease' may encompass a spectrum of conditions, from asymptomatic angina to myocardial infarction; this was not often specified. Such potential measurement errors would likely dilute effects in the meta-analysis. Third, we did not consider ocular factors (e.g., presence of large drusen), that have been found to be strong predictors of progression to late AMD, as we felt they could not be easily ascertained by family physicians. Neither did we consider dietary factors, such as the consumption of vegetables rich in carotenoids (lutein and zeaxanthin) or zinc and antioxidant vitamin supplementation, fish or omega-3 fatty acids in the diet, or glycemic index, because the methods for estimating such risk factors in the setting of primary health care is difficult without access to specialised personnel and resources [6,87-89]. Fourth, despite strong associations of AMD with genetic factors, we felt that genetic testing was not readily available in general clinical practice. Fifth, we included only English language articles because a preliminary assessment did not identify any non-English language articles that fitted our inclusion criteria. Nonetheless, many studies included in this review were from non-white populations (e.g., Chinese, Malay Asians) and thus, we believe our results can be generalisable to different populations in different countries around the world. Sixth, we only included articles that were identified in the Medline and Cochrane databases. Expanding the search to EMBASE may have identified additional articles; however, given the extensive hand searching of bibliographies and the experience of the authors we feel it is unlikely any relevant articles were missed. Seventh, the assessment of the quality of the publications was performed as part of this study to provide supplementary evidence of the internal and external validity of the data. However, ultimately, we decided to present the data based on the type of study design reported in the publication: cohort, cross sectional and case control. Large, epidemiological, cohort studies had the advantage over other study designs in that they removed any temporal or causal ambiguity as the exposure precedes the disease and if follow-up is not biased selection bias is less of a problem than in other study designs [90]. Finally, whether the study findings could be used as prognostic information to refer patients with higher risk of AMD requires further research. The observed odds ratios were generally small, and there are limited interventions to prevent AMD.

## Conclusions

Our systematic review and meta-analysis identified four strong and consistent risk factors for late AMD (increasing age, current smoking, previous cataract surgery and an AMD family history) and a further four risk factors showing significant and moderate strength of associations (high BMI, history of cardiovascular disease, hypertension and plasma fibrinogen). This study provides additional information for primary care physicians, general ophthalmologists and other eye care professionals to counsel their patients on AMD risk.

## Competing interests

Drs. Chakravarthy, Wong, Fletcher, and Mitchell were paid consultants to Pfizer Inc and Ms Piault and Dr. Evans were employees of Mapi Values, Boston, Massachusetts at the time the research was conducted and the manuscript was developed. Mapi Values is a consultancy whose activities on the project were funded by Pfizer. Drs. Zlateva and Pleil are employees of Pfizer Inc. Dr. Buggage was an employee of Pfizer during part of the study and now is an employee of Novartis, East Hanover, NJ. Usha Chakravarthy has served on advisory boards to Pfizer, Oraya Therapeutics, Allergan and Novartis and has received honoraria and travel support. She has been and continues to be an investigator on controlled clinical trials and observational studies sponsored by Pfizer, Novartis, Alcon and Bausch and Lomb and her department has received funds for the conduct of these studies.

Tien Wong has been on advisory boards for Pfizer, Allergan and Novartis, and has received honoraria and travel and accommodation payments from them. He has also received research support from Pfizer. He has also been an investigator on clinical trials sponsored by them and has received payments to support the conduct of these trials.

Astrid Fletcher has been on advisory boards for Pfizer and has received honoraria and travel and accommodation payments from them.

Elisabeth Piault and Chris Evans are employed by Mapi Values that has received research support from Pfizer. They have not been an investigator on clinical trials sponsored by them and has not received payments to support the conduct of any trials

Gergana Zlateva and Andreas Pleil are paid employees of Pfizer and have no other conflicts.

Paul Mitchell has been on advisory boards for Novartis, Pfizer, Allergan and Solvay, and has received honoraria and travel and accommodation payments from them. He has also been an investigator on clinical trials sponsored by these companies, as well as Lilly and Bayer, and has received payments to support the conduct of these trials.

## Authors' contributions

UC, TW, AF, EP, CE, GZ, RB, AP, PM participated in the design and review of the systematic research; EP and CE managed the data collection. EP, CE GZ, RB, AP provided technical expertise for the analysis and interpretation of the data. UC, TW, AF, PM provided clinical and technical expertise for the analysis and interpretation of the data. All authors (UC, TW, AF, EP, CE, GZ, RB, AP, PM) were involved with the manuscript development, and reviewed and approved the final version of the manuscript. AU and TW had full access to all the data in the study and take responsibility for the integrity of the data and the accuracy of the data analysis.

## Pre-publication history

The pre-publication history for this paper can be accessed here:

http://www.biomedcentral.com/1471-2415/10/31/prepub
